# Antipsychotic Drugs in Prevention of Postoperative Delirium—What Is Known in 2020?

**DOI:** 10.3390/ijerph17176069

**Published:** 2020-08-20

**Authors:** Michał P. Pluta, Magdalena Dziech, Piotr F. Czempik, Anna J. Szczepańska, Łukasz J. Krzych

**Affiliations:** 1 St. Barbara’s Memorial Hospital No. 5 Trauma Center, 41-200 Sosnowiec, Poland; michal_p2@o2.pl; 2 Students’ Scientific Society, Department of Anaesthesiology and Intensive Care, Faculty of Medical Sciences in Katowice, Medical University of Silesia, 40-752 Katowice, Poland; magdalena.dziech@gmail.com; 3 Department of Anaesthesiology and Intensive Care, Faculty of Medical Sciences in Katowice, Medical University of Silesia, 40-752 Katowice, Poland; pczempik@sum.edu.pl (P.F.C.); a.j.szczepanska@gmail.com (A.J.S.)

**Keywords:** postoperative delirium, prevention, perioperative medicine, antipsychotics

## Abstract

Delirium is one of the most frequently reported neuropsychiatric complications in the perioperative period, especially in the population of elderly patients who often suffer from numerous comorbidities undergoing extensive or urgent surgery. It can affect up to 80% of patients who require hospitalization in an intensive care setting postoperatively. Delirium increases mortality, morbidity, length of hospital stay, and cost of treatment. An episode of delirium in the acute phase may lower the general quality of life and increases the risk of cognitive decline long-term. Since pharmacological treatment of delirium is not highly effective, focus of research has shifted towards developing preventive strategies. We aimed to perform a review of the topic based on the most recent literature. We conclude that, based on the available data, it seems impossible to make strong recommendations for using antipsychotic drugs in prophylaxis. Further research should answer the question what, if any, benefit patients receive from the pharmacological prevention of delirium, and which agents should be used.

## 1. Introduction

Delirium is a set of neuropsychiatric symptoms, including qualitative disturbances of consciousness and attention, which may be accompanied by cognitive deficits and psychotic symptoms [1]. It is characterized by an acute onset with fluctuations of symptom intensity throughout the day, reaching its apogee usually in the evening and night [2]. Due to the spectrum of observed symptoms, delirium may take hypoactive, hyperactive, or mixed form [3].

The incidence of delirium varies from several percent in patients hospitalized in general wards up to 80% in patients with multi-organ failure treated in intensive care units (ICU) [4,5]. Hypoactive delirium in the most frequent form, accounting for almost half of all delirium cases and may not be recognized even in 80% of patients, due to the coexistence of dementia or use of sedative drugs, which cannot always be discontinued in the ICU [6,7].

Diagnosis of postoperative delirium (POD) should be based on updated criteria in the Diagnostic and Statistical Manual of Mental Disorders (DSM) [1]. Although it is a gold standard diagnostic tool, it can be difficult to use even by experienced non-psychiatrists [8]. Therefore, screening for delirium may be more effective when using validated diagnostic scales: Confusion Assessment Method for the Intensive Care Unit (CAM-ICU) [9,10], Intensive Care Delirium Screening Checklist (ICDSC) [11], Delirium Observation Screening (DOS) [12], Neelon and Champagne Confusion Scale (NEECHAM) [13], or others, which have found popularity in scientific research regarding delirium. These methods are summarized in Table 1.

**Figure 1:**
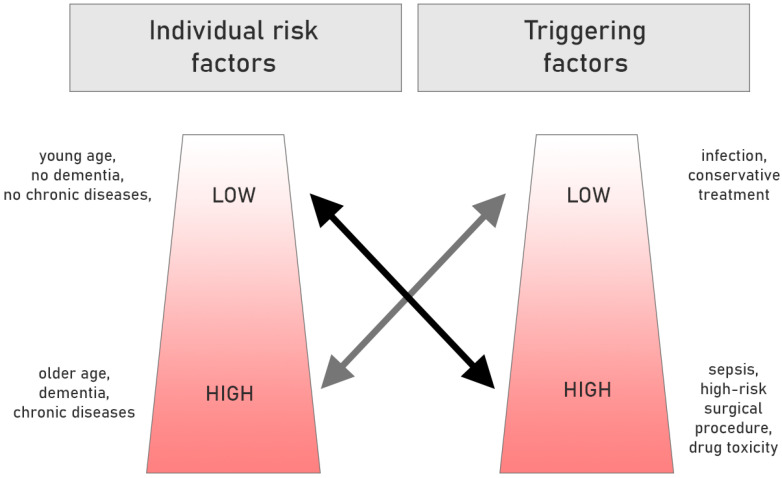
The role of predisposing and triggering factors in the occurrence of delirium. Based on [15,16] (with modification).

Risk factors for delirium can be divided into predisposing (mainly demographic variables and conditions related to the burden of additional diseases and the degree of their control) and precipitating (triggering) (these are primarily fluctuations in the current clinical state, associated with current hospitalization or therapy). The greater the burden of predisposing factors, the lower the exposure to triggers is likely to cause delirium, and vice versa. Figure 1 illustrates these connotations.

Elderly patients with numerous comorbidities are particularly susceptible to delirium. Other POD risk factors are: suboptimal pain control, inadequate opioid analgesia and the use of meperidine in elderly patients, intra- and postoperative hypotension and hypoperfusion, anemia, independent of the need for transfusion, malnutrition, acid-base and water-electrolyte balance disorders, deprivation of sleep, severe infections, and chronic psychiatric disorders (depression, dementia, cognitive deficits) [17,18,19,20]. Among this diversity of variables, it is important to remember that delirium is favored by all states of impaired cerebral flow and hypoxia, as well as intoxications that interfere with the metabolism of neurotransmitters. The listed risk factors differ between populations, fields of medicine, and treatment specialties. Due to the fact that some of these risk factors can be modified, optimizing the patient’s clinical condition well in advance can reduce the baseline risk of POD.

Surgical procedure, especially in a non-elective surgery, general anesthesia, prolonged mechanical ventilation, in turn, are factors that can trigger delirium in susceptible patients [21].

The association between delirium and increased mortality was reported previously [22]. The long-term consequences of POD having negative impact on patients’ social functioning include dementia, deterioration of quality of life, depression, mood disorders, post-traumatic stress disorder, dependence on third-party help, and loss of earning potential [23]. It is also estimated that the cost of treating patients with delirium in the United States alone is on the order of tens of millions of US dollars each year [24,25].

POD pathophysiology has not been fully explained [26]. Theories based on neurotransmitter disorders, mainly acetylcholine and dopamine, have been confirmed in pathophysiological models and have become a potential avenue for pharmacological research [27]. Up-to-date studies suggest that the use of pharmacological agents after the onset of full-symptom POD is ineffective, and may even lead to longer duration and severity of delirium, so more attention should be given to prevention [28]. Medical activities focus primarily on relieving delirium symptoms. The beneficial effect of pharmacotherapy tends to be seen more often when introduced prophylactically or at the stage of sub-symptomatic delirium (SSD, subsyndromal delirium), which occurs in the period between the absence of symptoms and the development of full-blown POD [29]. However, these hypotheses need to be verified in further research due to conflicting conclusions. Fighting and controlling predisposing factors and avoiding delirium triggers remains the golden rule, although this is not always possible [30].

Just as POD etiology is multifactorial, its prevention and treatment should be multifaceted [30]. Before pharmacological treatment, non-pharmacological interventions constitute a mandatory preventive element [31,32]. These include pre-rehabilitation, improving patient orientation, early mobilization after surgery, providing conditions for natural sleep and rest, contact with family, and proper nutrition [33].

Studies on the pharmacological prevention of delirium have so far focused on acetylcholinesterase inhibitors, melatonin, ketamine, clonidine, ondansetron, statins, and corticosteroids [34,35,36,37]. A recent network meta-analysis showed decrease in the occurrence of delirium with ramelteon (MT1 and MT2 melatonin receptor agonist) [38]. A reduction in the frequency of POD has also been shown in patients preventively receiving dexmedetomidine [34]. However, to this day, little attention was given to antipsychotic drugs in the surgical population [39].

Although there is no convincing evidence for the efficacy of haloperidol in the treatment of delirium, it is often used in ICUs [40]. As a typical antipsychotic drug, it blocks dopaminergic receptors, acts as an anticholinergic agent, and has affinity for serotonin receptors [41]. It has good bioavailability after oral administration, but intravenous supply is preferred in the population of critically ill patients, whose safety, despite the lack of registration (off-label), has been confirmed in many studies [42,43]. Interesting reports of Levenson on intravenous use of haloperidol in a daily dose of 1155 mg and a total four-day dose of 2842 mg have been published, however, they should be approached with extreme caution [44].

Adverse effects of haloperidol include extrapyramidal disorders (Parkinson’s syndrome, acute dyskinetic syndrome), hyperthermia, seizures, and neuroleptic malignant syndrome [41]. QT prolongation may promote ventricular arrhythmias [45]. Second-generation atypical antipsychotics, such as quetiapine, olanzapine, risperidone, and ziprasidone, are less likely to cause adverse motor effects due to their affinity for 5HT2_A_ serotonin receptors [46,47,48,49]. Atypical neuroleptics, however, are not available in parenteral form, which may limit their use. A brief description of antipsychotic drugs is presented in Table 2.

## 2. Methods

We performed a comprehensive e-based search for randomized clinical trials conducted in the surgical patient population, published in English in PubMed, Embase, and the Cochrane Library by June 2020. The search strategy included the following key terms: delirium, emergence delirium, POD, cognitive defect, cognitive dysfunction, cognitive impairment, ACS, acute confusional state, POCD, confusion, hallucination, agitation, neurocognitive disorders, postoperative, antipsychotic drug, risperidone, olanzapine, quetiapine, ziprasidone, haloperidol. Firstly, the abstracts were analyzed by two independent researchers. Then, after obtaining a consistent opinion, full versions of papers were retrieved and scrupulously analyzed by the team of four researchers. Additionally, the full papers were verified manually to identify additional studies requiring inclusion. Due to the small number of studies and significant methodological differentiation, the attempt to perform a systematic review and meta-analysis was abandoned.

## 3. Prevention of Delirium

### 3.1. Haloperidol

One of the first attempts to evaluate the use of haloperidol in POD prevention was described by Kaneko et al. [42]. In their study 78 patients underwent gastrectomy (partial or total) or colectomy. The intervention group (n = 38, mean age 72 ± 8 years) received intravenous haloperidol at a dose of 5 mg (at 21:00) from one to five days after surgery. The control group (n = 40, mean age 73 ± 9 years) received placebo. POD, diagnosed based on DSM-III-R criteria, occurred in 22% of patients, significantly less frequently in patients receiving haloperidol (10.5 vs. 32.5%; RR = 0.32; 95% CI 0.12–0.91; *p* < 0.05). In the POD control group, it lasted longer and was more severe, although the authors did not provide criteria for assessing the severity of delirium. Sleep and wakefulness disturbances were observed in patients who developed POD, including reduction in nighttime sleep at the cost of greater daytime sleepiness. The authors failed to demonstrate a relationship between postoperative pain control methods, hypoxia episodes and infection, and the occurrence of POD. No adverse effects were reported in patients receiving haloperidol.

In the study of Kalisvaarta et al. [50] haloperidol was used in patients over 70 years of age who underwent elective or urgent hip surgery. The study included patients with intermediate and high risk POD, assessed on the basis of four criteria: visual impairment, severity of disease (i.e., Acute Physiology and Chronic Health Evaluation II > 15 points) and cognitive impairment (i.e., Mini-Mental State Examination Score < 25 points), and dehydration index (blood urea nitrogen to creatinine ratio > 17). An intermediate risk was the presence of one or two criteria (179 patients in the intervention group and 181 in the control group), and high risk was found when three or four of them were met (33 patients in the intervention group and 35 in the control group). From the analysis have been Low-risk patients; previously diagnosed with delirium, deep dementia, Parkinson’s disease, epilepsy; intubated, treated with acetylcholinesterase inhibitors and patients in whom haloperidol was contraindicated (sensitization, QT prolongation > 460 ms). The study group consisted of 430 patients. The intervention group (n = 212) received oral haloperidol (0.5 mg every eight hours) on the day of surgery and for three consecutive days. Placebo was administered in the control group (n = 218). The incidence of delirium, based on DSM-IV and CAM criteria, was comparable in the intervention and control groups (15.1 vs. 16.5%; RR = 0.93; 95% CI 0.6–1.4). However, haloperidol reduced the severity of symptoms, rated as the highest on the DRS-R-98 scale (14.4 ± 3.5 vs. 18.4 ± 4.4; *p* < 0.001). The duration of POD was also shorter in the intervention group (5.4 vs. 11.8 days; *p* < 0.001). POD was more common in high-risk patients (12%; 95% CI 8.7–15.3) compared to the intermediate risk group (38%; 95% CI 26.1–51.2). Preventive use of haloperidol reduced the time to rehabilitation ward or home discharge by an average of 5.5 days (95% CI 1.4–2.3; *p* < 0.001). No adverse effects related to drug administration have been reported.

Interesting data was provided by a prospective cohort study, conducted by Vochteloo et al. [51], who analyzed patients aged 65+ undergoing surgical treatment for hip fracture. They implemented the single-center POD prophylaxis standard (SOP), including the original screening model RD (Risk Model of Delirium) developed on the basis of retrospective data. The result of <5 points in the RD model was characterized by a large (86%), negative predictive value for delirium recognition, with a low (42%) positive predictive value. The study was conducted in a group of 378 patients. Patients with a score of ≥5 points (n = 173) were at high risk for POD (OR 4.13; 95% CI 2.43–7.02) and received prophylactic haloperidol (1mg every 12 hours orally). The low risk group (n = 205) received a placebo. The surgery was performed under conduction anesthesia in 97.5% of high-risk patients and 91.1% of low-risk patients, respectively. The frequency of POD, recognized on the basis of DSM-IV criteria, was 27% in the studied cohort (42% in the high risk group and 14% in the low risk group). The implementation of SOP in patients with a hip fracture did not reduce the incidence of POD compared to historical groups (2005: 29%, *p* = 0.3; 2006: 24%, *p* = 0.8; 2007: 28%, *p* = 0.44).

Wang et al. [43] demonstrated benefit of the prophylactic use of haloperidol in patients over 65 years of age undergoing non-cardiac surgery. Exclusion criteria included preoperative history of schizophrenia, epilepsy, parkinsonism, the use of acetylcholinesterase inhibitors and neuroleptics, dementia, neurosurgical procedures, and contraindications for haloperidol (allergy, QT prolongation). The intervention group (n = 229) received a haloperidol bolus (0.5 mg intravenously) after transfer to the ICU, followed by a continuous infusion (0.1 mg h^−1^) for 12 hours up to a total daily dose of 1.7 mg. Such pharmacological prophylaxis reduced the incidence of delirium in the first 7 days of hospitalization in the ICU (15.3% in the haloperidol group vs. 23.2% in the placebo group; RR = 0.66; 95% CI 0.45–0.97; *p* = 0.03). There were no differences in 28-day mortality between intervention and control groups (0.9 vs. 2.6%; *p* = 0.2). No serious adverse effects were reported.

The open, randomized study of Fukata et al. [52] included patients over 75 years of age who underwent orthopedic or gastroenterological elective surgery. The intervention group received haloperidol intravenously at a dose of 2.5 mg every 24 hours for three days after discharge from the operating theater. The control group was given a placebo. POD (NEECHAM <20) occurred in 38% of patients. Preventive administration of haloperidol did not reduce the incidence of POD within seven days after surgery (42% in the intervention group vs. 33% in the control group; *p* = 0.3). The positive effect of haloperidol in reducing the frequency of POD was also not demonstrated in the subgroup of high-risk patients, defined as the result of 25–27 points according to NEECHAM (53% in the intervention group vs. 43% in the control group; *p* = 0.9) or <25 points according to MMSE (67% in the intervention group vs. 64% in the control group; *p* = 0.4).

In the next study, the same authors [53] used an increased dose of haloperidol (5 mg intravenously) in 201 patients aged 75+ undergoing surgery, abdominal and orthopedic surgery, under general or regional anesthesia. Prophylaxis was used from zero to five days after surgery. Severe POD (NEECHAM <19) was less common in the haloperidol group (18.2 vs. 32%; *p* = 0.03). Haloperidol did not reduce the duration of POD (median: two days in both groups; *p* = 0.45) and length of hospitalization (median: 16 days in both groups; *p* = 0.3) in patients who developed delirium despite prophylaxis. The limitation of the research by Fukata et al. [51,52] was the lack of double blindness. Regardless of the results obtained, no serious adverse effects of haloperidol were observed in both studies.

Van den Boogaard et al. [54] conducted a multicenter, double-blind RCT, in which they evaluated the use of haloperidol (2 mg every eight hours intravenously) in patients at high risk of delirium (expected hospitalization in ICU > 2 days), admitted to ICUs in 21 Dutch centers. The drug was administered within the first 24 hours of admission into an ICU and continued until discharge from the ward or diagnosis of delirium, but no longer than 28 days. In all centers, non-pharmacological delirium prevention methods were routinely used at the same time, including early mobilization, circadian rhythm optimization, noise reduction, sedation and benzodiazepine use, and hearing aids. In the subanalysis of surgical patients (n = 828), there were no differences in the delirium frequency (41.2 vs. 37.2%; OR = 1.19, 95% CI 0.87–1.62, *p* = 0.4) and 28-day survival (87.5 vs. 86.9%; HR = 0.95, 95% CI 0.62–1.45, *p* = 0.8) between the intervention and control groups. However, the authors did not provide a broader analysis of demographic and clinical data (including haloperidol adverse reactions) for the surgical subpopulation. Screening for POD was conducted for 28 days of hospitalization in the ICU, but transferring a patient to another ward was seen arbitrarily as a delirium, which may underestimate late-onset delirium episodes.

The studies are presented in Table 3.

An interesting summary of clinical observations was the meta-analysis of Lin et al. [39], which showed that prophylactic use of haloperidol in patients admitted to the ICU after surgery can reduce the frequency of POD (3 RCT, n = 670; RR 0.63; 95 % CI 0.47–0.86; *p* < 0.001). However, no beneficial effect was demonstrated when prophylaxis was routinely used in all patients treated in the ICU without considering the reason for hospitalization (6 RCT, n = 1567; RR 0.66; 95% CI 0.62–1.1; *p* = 0.2) suggesting that the subpopulation of surgical patients may require different management. Pharmacological prophylaxis had no effect on reducing mortality (5 RCT, n = 2159; RR = 0.59; 95% CI 0.76–1.18; *p* = 0.6) or increasing the risk of haloperidol side effects, including prolongation of the QT interval (5 RCT, n = 1806; RR = 1.01; 95% CI 0.64–1.59; *p* = 0.98), arrhythmias (3 RCT, n = 652; RR = 1.05; 95% CI 0.56–1.97; *p* = 0.9), and extrapyramidal symptoms (5 RCT, n = 1824; RR = 0.62; 95% CI 0.35–1.09; *p* = 0.1).

On the other hand, Shen et al. [55] performed a meta-analysis of studies including different dosages of haloperidol in POD prophylaxis. Based on seven RCTs, a daily intravenous dose of haloperidol ≥5 mg has been shown to be more effective in preventing POD (2 RCT, n = 279; RR 0.50; 95% CI 0.32–0.79; *p* = 0.003) compared to lower doses (5 RCT, n = 1314; RR 0.96; 95% CI 0.73–1.28); *p* = 0.8). Prophylaxis with higher doses of haloperidol requires observation for extrapyramidal symptoms and QT prolongation, because the frequency of these disorders increases with dose (QTc prolongation: 1.8% vs. 9.9%; extrapyramidal symptoms: 0% vs. 2, 8% for 1.7 mg per day and 7.5 mg per day respectively [43,52].

### 3.2. Atypical Antipsychotics

The use of risperidone after elective cardiac surgery using extracorporeal circulation was studied by Prakanrattana et al. [56]. The intervention group (n = 63; mean age 61.3 ± 9.7 years) received 1 mg of risperidone sublingually once after awakening in the postoperative ward. Compared to the placebo control group (n = 63; mean age 60.7 ± 9.8 years), the incidence of POD in the intervention group was significantly lower (31.7 vs. 11.1%; RR = 0.35; 95% CI 0.16–0.77; *p* = 0.009). In both groups, PODs were most often diagnosed within the first day after surgery. A relationship has been demonstrated between postoperative respiratory failure (19 vs. 1%, *p* = 0.002 in the delirium and non-delirium group, respectively), renal failure (15 vs. 1%, *p* = 0.007) and prolonged time from eye opening to compliance (112 vs. 62 min, *p* = 0.002), and the occurrence of POD. Patients who were delirious stayed in the ICU nearly twice as long (4.7 vs. 2.8 days; *p* = 0.002). The total hospital stay in the POD group was also longer (13.3 vs. 9.6 days; *p* = 0.004).

Hakim et al. [29] evaluated the effectiveness of risperidone in patients with SSD. The study included 101 patients aged 65+ who were diagnosed with SSD after cardiac surgery with extracorporeal circulation. SSD assessments were made four hours after extubation in a cardiac surgery ICU and repeated every 8 hours. In the case of SSD diagnosis, defined as 1–3 points on the ICDSC scale, randomization was performed. The intervention group (n = 51) received 0.5 mg risperidone orally every 12 hours. Risperidone was discontinued 24 hours after resolution of SSD (0 points according to ICDSC) or on suspicion of delirium (≥4 points according to ICDSC). Compared to the placebo control group (n = 50), risperidone reduced the incidence of POD (34 vs. 13.7%; *p* = 0.031). It was shown that SSD under treatment was an independent risk factor for the development of full-blown delirium (HR = 3.83; 95% CI 1.63–8.98; *p* = 0.002). No adverse effects of risperidone have been reported.

The efficacy of olanzapine in POD prophylaxis has been assessed in only one double-blind RCT, involving 400 patients aged 65+ undergoing elective orthopedic knee or hip replacement surgery [28]. The study excluded patients with dementia, actively consuming more than 10 drinks per week, or alcohol addicts, taking antipsychotics, and reporting allergic reactions to olanzapine. In the intervention group (n = 196), patients received 5 mg of olanzapine before surgery and 5 mg after transfer to the operating room. Placebo was used in the control group (n = 204). POD assessments were made using DSM-III-R criteria for eight days after surgery, and when diagnosed, POD severity was assessed using the DRS-R-98 scale. The study protocol assumed the intravenous supply of 1–2 mg pre-medicated midazolam. 90% of patients underwent general complex anesthesia and the remaining 10% spinal or epidural anesthesia. The protocol of postoperative analgesia was not uniform for the entire study group. It anticipated regional analgesia (blockage of the femoral nerve after knee replacement surgery) in selected patients, and in patients with epidural anesthesia, continued bupivacaine infusion in the postoperative ward. In other patients, on intravenous opioids were used. To sum up, the study showed a relationship between prophylactic use of 10 mg olanzapine and a reduction in the incidence of POD in the entire study population (14.3 vs. 40.2%; RR = 0.35; 95% CI 0.24–0.52; *p* < 0.001), as well as in subgroups of patients qualified for knee (17.7 vs. 47.8%; RR = 0.37; 95% CI 0.24–0.56; *p* < 0.0001) and iliac (7.6 vs. 30.8%; RR = 0.24; 95% CI 0.10–0.60; *p* < 0.0004) surgery. However, with POD, it lasted longer in patients receiving prophylactic olanzapine (2.2 vs. 1.6 days; *p* = 0.02) and was more severe, expressed as the highest DRS-R-98 score on day 1 of delirium (16.4 vs. 14.5 points; *p* = 0.02). The authors noted significant difference between the intervention and control groups in the baseline albumin concentration [24,28]. Since olanzapine is 93% bound to plasma proteins, mainly albumin and alpha-1 acid glycoprotein, hypoalbuminemia may have impacted the effectiveness of pharmacological prophylaxis [57]. Reduced albumin concentration is also an independent risk factor for delirium [58]. Assessment of the impact of prophylactic use of olanzapine on the severity of POD requires verification in subsequent studies.

The studies are summarized in Table 4.

### 3.3. Haloperidol vs. Atypical Antipsychotics

Meta-analysis Fok et al. [59] showed that atypical antipsychotics reduce the risk of POD more effectively (OR = 0.252; 95% CI 0.163–0.389; *p* = 0.000) than haloperidol (OR = 0.599; 95% CI 0.408–0.822; *p* = 0.009), but the final effect probably only benefits high-risk patients.

There are also reports in the literature that prove the ineffectiveness of the pharmacological prophylaxis of POD with antipsychotic drugs, both typical and second generation neuroleptics [60]. This shows that the problem is unresolved on a scientific level. Therefore, transferring the above observations into clinical practice must be approached with caution.

## 4. Limitations

We currently lack sufficient data on pharmacological prophylaxis of POD using antipsychotic drugs. As the analysis of the included works shows, the studies carried out so far were characterized by considerable heterogeneity in relation to the patient populations, types of surgeries, anesthesia, and modes of therapy. Most often these studies included elderly patients. Although this is the most vulnerable population, POD can occur in all age categories. Research was conducted primarily in cardiac surgery, orthopedics, and abdominal surgery. However, the spectrum of procedures is much wider. The comparison of research results is made difficult by discrepancies in the scope of drug doses, route of administration, method of administration (bolus or continuous infusion), time of prophylaxis (before or after surgery), frequency, and time of taking medication and the total dose of neuroleptic administered in the perioperative period. The oral route of treatment may not be optimal in selected groups of patients, e.g., those undergoing gastrointestinal surgery. In addition, both routes may show significant differences in drug bioavailability [53]. Zhang et al. attempted to standardize the dosage by converting each study drug into an equivalent oral dose of haloperidol, but this effect is at high risk of error, especially in patients hospitalized in the ICU postoperatively [24]. Only two studies contained information on non-pharmacological prophylaxis methods for POD. We have not identified studies that directly compared the efficacy of haloperidol with second-generation drugs, as well as oral and intravenous haloperidol in surgical patients. Available works mainly focus on the treatment of critically ill patients and the ICU environment. In some of them, haloperidol has been shown to have similar efficacy in the treatment of delirium as atypical neuroleptics [55]. In another study, there was no reduction in the duration of delirium with haloperidol or ziprasidone in patients hospitalized in the ICU with delirium and acute respiratory failure or shock [61]. Reports differed in the context of the accepted method of delirium recognition and assessment of the severity of symptoms. Despite the data on greater efficacy of pharmacological prophylaxis in high risk POD groups, we do not currently have a sufficiently good predictive tool to identify patients at risk who may benefit from the administration of antipsychotic drugs [59]. Large discrepancies between studies relate to the inclusion and exclusion criteria adopted by the authors. In two projects, alcohol dependence was used as the exclusion criterion, but the threshold value of a declared, weekly dose of alcohol differed between the authors [28,50]. This heterogeneity in defining alcohol dependence could have impact on a potential factor affecting the supposed severity of delirium in patients receiving prophylactic olanzapine in the study of Larsen et al. [24,28]. For the treatment of withdrawal delirium, the use of benzodiazepines [62] is recommended. The authors also did not present the criteria for admitting patients in the ICU in the postoperative period. It is difficult to imagine that this was a routine procedure for every patient undergoing surgery. In turn, the initial reason for admission in the ICU could significantly modify the initial POD risk. The remaining limitation of the study was a lack of reference for the effects of preventive measures applied during the intraoperative period. The long duration of the procedure, hemodynamic instability or sudden blood loss requiring transfusion may be the factors that increase the risk of POD [63]. In turn, a reduced risk of delirium has been reported in patients in whom the depth of anesthesia was monitored intraoperatively using a bispectral index [64]. The study groups differed significantly in terms of how postoperative analgesia was performed, what could also affect the results [65]. Janssen et al. proved that optimal control of postoperative pain can successfully reduce the frequency of POD episodes [33]. Current concepts of delirium prevention in intensive care (PAD, Pain-Agitation-Delirium and e-CASH, early Comfort using Analgesia, minimal Sedatives and maximal Human care) prioritize pain control, personalized patient care, and the use of non-pharmacological prevention methods [66,67]. From the studies mentioned, little can be deduced about non-pharmacological methods: whether they should be used, and if so, which of them and to what extent?

Finally, a significant limitation of the conducted studies was the lack of evaluation of the impact of pharmacological prophylaxis on the development of various types of POD, especially the hypoactive form. Although there is evidence that the response to treatment with haloperidol in the form of hypo- and hyperactive POD is similar [68], it is not possible to draw conclusions regarding prophylaxis with neuroleptics.

## 5. Conclusions

Currently, based on the available data, no recommendations can be made for the routine prophylaxis of postoperative delirium with antipsychotic drugs. However, some studies have shown the benefit of the prophylactic use of neuroleptics with a minimal risk of serious side effects, opening the way for further research to answer the question which patients can benefit from the pharmacological prevention of delirium and how to use these agents effectively based on high-quality evidence. The potential role of dexmedetomidine in POD prevention cannot be neglected.

## Figures and Tables

**Table 1:** Criteria for the diagnosis of delirium in scientific research.

Method	Diagnostic Criteria
DSM-V * [1]	Disturbance of consciousness (i.e., reduced clarity of awareness of the environment) reduced ability to focus, sustain, or shift attention. Change in cognition (e.g., memory deficit, disorientation, language disturbance, perceptual disturbance) that cannot be better accounted for by a preexisting, established, or evolving dementia. The disturbance develops over a short period of time (usually hours to days) and tends to fluctuate during the course of the day. Evidence from the history, physical examination, or laboratory findings indicates that the disturbance is caused by a direct physiologic consequence of a general medical condition, an intoxicating substance, medication use, or more than one cause.
CAM (CAM-ICU) [9,10]	If features 1 and 2 and either 3 or 4 are present (CAM +/positive), a diagnosis of delirium is suggested: Acute onset and fluctuating course (1) Inattention (2) Disorganized thinking (3) Altered level of consciousness (4)
ICDSC [11]	Altered level of consciousness Inattention Disorientation Hallucinations or delusions Psychomotor agitation or retardation Inappropriate speech or mood Sleep/wake cycle disturbance Symptom fluctuation
DOS [12]	Dozes off during conversation or activities Is easily distracted by stimuli from the environment Maintains attention to conversation or action Does not finish question or answer Gives answers that do not fit the question Reacts slowly to instructions Thinks they are somewhere else Knows which part of the day it is Remembers recent events Is picking, disorderly, restless Pulls intravenous tubing, feeding tubes, catheters, etc. Is easily or suddenly emotional Sees/hears things which are not there Responses are dichotomous (yes/no). Scores ≥ 3 were considered positive delirium screens
DRS [14]	Temporal onset (three points) Perceptual disturbances (three points) Hallucinations (three points) Delusions (three points) Psychomotor (three points) Cognition (four points) Physical etiology (two points) Sleep-wake cycle (four points) Mood lability (three points) Fluctuation (four points) Total scores of ≥12–diagnosis of delirium
NEECHAM [13]	Level of responsiveness-information processing ⚬ Attention and alertness (0–4 points) ⚬ Verbal and motor response (0–5 points) ⚬ Attention and alertness (0–5 points) Level of behavior ⚬ General behavior and posture (0–2 points) ⚬ Sensory motor performance (0–4 points) ⚬ Verbal responses (0–4 points) Vital functions ⚬ Vital signs (0–2 points) ⚬ Oxygen saturation level (0–2 points) ⚬ Urinary continence (0–2 points) 0–19 points: moderate to severe confusion 20–24 points: mild or early development of delirium 25–30 points: not confused or normal function

* Gold standard.

**Table 2:** Characteristics of antipsychotic drugs.

Drug	Mechanism	Initial Dose	Selected Side Effects
Haloperidol [41]	Competitively blocks postsynaptic dopamine (D_2_) receptors in the mesolimbic system of the brain; blocks cholinergic and histaminergic receptors	1–5 mg po./im./iv.	Parkinsonism, akathisia, dystonia, tardive dyskinesia, QT-prolongation, sedation, neuroleptic malignant syndrome
Risperidone [46]	Selective serotonin antagonist (cortical 5-HT_2_ receptor), competes with dopamine at the limbic dopamine (D_2_) receptor	0.5–1 mg po.	
Quetiapine [47]	Serotonin antagonist (5-HT_1_, 5HT-_2_ receptors); reversibly binds to dopamine (D_1_, D_2_) receptors in the mesolimbic and mesocortical areas	25–50 mg po.	
Olanzapine [48]	Binds with high affinity binding to the serotoninergic (5HT_2_, 5HT-_3_), dopaminergic (D_2_), muscarinic (M_1_-M_5_), histamine (H_1_), and alpha-1-adrenergic receptors	5–10 mg po.	
Ziprasidone [49]	Antagonist dopamine (D_2_), serotonin (5-HT2A, 5-HT1D), histamine (H1) and alpha-1-adrenergic receptors, agonist 5-HT1A receptor	2.5–5 mg po.	

**Table 3:** Studies evaluating the effectiveness of haloperidol in the prevention of POD.

First Author, Year, Type of Study	Type of Surgery	Mean Age	Antypsychotics	Dose	POD (%)	Outcome (Frequency POD)
Kaneko et. al., 1999, RCT [45]	GI surgery	72 ± 8	Haloperidol (vs. placebo)	5 mg iv. on postoperative days 1–5	22%	OR = 0.24; 95% CI 0.07–0.84
Kalisvaart et. al., 2005, RCT [50]	Orthopedic surgery	78 ± 6	Haloperidol (vs. placebo)	0.5 mg po. preoperative and for 3 days after surgery	16%	No benefit
Vochteloo et al., 2011, PCT [51]	Orthopedic surgery	87 ± 6	Haloperidol	1 mg po. every 12 h	27%	No benefit
Wang et al., 2012, RCT [43]	Mixed	74 ± 6	Haloperidol (vs. placebo)	0.5 mg iv. postoperative and infusion 0.1 mg h^−1^ for 12 h	19%	OR = 0.60; 95% CI 0.37–0.96
Fukata et al., 2014, Randomized open-label prospective trial [52]	Abdominal or orthopedic surgery	80.5 ± 0.5	Haloperidol (vs. placebo)	2.5 mg iv. every 24 hours for 3 days after surgery	38%	No benefit
Fukata et al., 2017, Randomized open-label prospective trial [53]	Abdominal or orthopedic surgery	82 ± 4	Haloperidol (vs. placebo)	5 mg iv. every 24 h for 5 days after surgery	25%	OR = 0.39; 95% CI 0.17–0.87
Van den Boogaard et al., 2018, RCT [54] *	Mixed	no data	Haloperidol (vs. placebo)	2 mg iv. every 8 h until the end of hospitalization or POD diagnosis	32%	No benefit

RCT, randomized control trial; PCT, prospective cohort study; GI, gastrointestinal; * subanalysis of surgical patients

**Table 4:** Studies evaluating the effectiveness of atypical neuroleptics in the prevention of POD.

First author, Year, Type of Study	Type of Surgery	Mean Age	Antypsychotics	Dose	POD (%)	Outcome (Frequency POD)
Prakanrattana et al., 2007, RCT [56]	Cardiac surgery	61 ± 10	Risperidone (vs. placebo)	1 mg p.o. after surgery	21%	OR = 0.27; 95%CI 0.10–0.69
Hakim et al., 2012, RCT [29] *	Cardiac surgery	65+	Risperidone (vs. placebo)	0.5 mg p.o. every 12 h	24%	OR = 0.31; 95%CI 0.11–0.83
Larsen et al., 2010, RCT [28]	Orthopedic surgery	73 ± 6	Olanzapine (vs. placebo)	5 mg p.o. before and after surgery	28%	OR = 0.25; 95%CI 0.15–0.40

RCT, randomized control trial; * prophylaxis in subsyndromal POD.
